# Development and validation of an UPLC–MS/MS assay for the simultaneous quantification of seven commonly used antibiotics in human plasma and its application in therapeutic drug monitoring

**DOI:** 10.1093/jac/dkae047

**Published:** 2024-02-28

**Authors:** Xin Meng Mekking, Kirsten Velthoven-Graafland, Marga J A Teulen, Roger J M Brüggemann, Lindsey H M te Brake, Nynke G L Jager

**Affiliations:** School of Pharmacy, Utrecht University, Leuvenlaan 4, 3584 CE, Utrecht, The Netherlands; Department of Pharmacy, Radboud Institute for Medical Innovation, Radboud University Medical Centre, Geert Grooteplein Zuid 10, 6525 GA Nijmegen, The Netherlands; Department of Pharmacy, Radboud Institute for Medical Innovation, Radboud University Medical Centre, Geert Grooteplein Zuid 10, 6525 GA Nijmegen, The Netherlands; Department of Pharmacy, Radboud Institute for Medical Innovation, Radboud University Medical Centre, Geert Grooteplein Zuid 10, 6525 GA Nijmegen, The Netherlands; Department of Pharmacy, Radboud Institute for Medical Innovation, Radboud University Medical Centre, Geert Grooteplein Zuid 10, 6525 GA Nijmegen, The Netherlands; Department of Pharmacy, Radboud Institute for Medical Innovation, Radboud University Medical Centre, Geert Grooteplein Zuid 10, 6525 GA Nijmegen, The Netherlands

## Abstract

**Objective:**

To develop and validate an UPLC–MS/MS assay for simultaneous determination of the total concentration of ceftazidime, ciprofloxacin, flucloxacillin, piperacillin, tazobactam, sulfamethoxazole, *N*-acetyl sulfamethoxazole and trimethoprim, and the protein-unbound concentration of flucloxacillin, in human plasma to be used for research and clinical practice.

**Methods:**

Sample pretreatment included protein precipitation with methanol. For the measurement of protein-unbound flucloxacillin, ultrafiltration was performed at physiological temperature. For all compounds, a stable isotopically labelled internal standard was used. Reliability of the results was assessed by participation in an international quality control programme.

**Results:**

The assay was successfully validated according to the EMA guidelines over a concentration range of 0.5–100 mg/L for ceftazidime, 0.05–10 mg/L for ciprofloxacin, 0.4–125 mg/L for flucloxacillin, 0.2–60 mg/L for piperacillin, 0.15–30 mg/L for tazobactam, 1–200 mg/L for sulfamethoxazole and *N*-acetyl sulfamethoxazole, 0.05–10 mg/L for trimethoprim and 0.10–50 mg/L for unbound flucloxacillin. For measurement of total concentrations, the within- and between-day accuracy ranged from 90.0% to 109%, and 93.4% to 108%, respectively. Within- and between-day precision (variation coefficients, CVs) ranged from 1.70% to 11.2%, and 0.290% to 5.30%, respectively. For unbound flucloxacillin, within-day accuracy ranged from 103% to 106% and between-day accuracy from 102% to 105%. The within- and between-day CVs ranged from 1.92% to 7.11%. Results of the international quality control programme showed that the assay is reliable.

**Conclusions:**

The method provided reliable, precise and accurate measurement of seven commonly prescribed antibiotics, including the unbound concentration of flucloxacillin. This method is now routinely applied in research and clinical practice.

## Introduction

Most antibiotics are dosed in a traditional, standard fixed-dose regimen, adjusted only in specific conditions such as renal failure, organ failure or extracorporeal life support. In the last decades, it has become clear that there is a large interindividual variability in pharmacokinetics, and thus exposure, of many antibiotics. Consequently, empiric approaches to antibiotic dosing may result in suboptimal antibiotic concentrations, possibly resulting in treatment failure or toxicity. Investigating antibiotic exposure in different patients using an accurate bioanalytical assay will help to better understand pharmacokinetic differences. The use of an accurate assay is also indispensable to perform therapeutic drug monitoring (TDM), in which dosing regimens are individualized based on measured drug concentrations. With TDM, risk of toxicity can be minimized while the chance of efficacy is maximized. The frequently prescribed antibiotics ceftazidime, ciprofloxacin, flucloxacillin, piperacillin/tazobactam, sulfamethoxazole and trimethoprim are known to have a large interindividual variability in exposure.

Traditionally, total drug concentrations are measured and unbound concentrations are calculated using protein-binding values from literature or predicted using models.^1–7^ This method is likely to be reliable for drugs with relatively low protein binding (<80%), such as ceftazidime (±20%), ciprofloxacin (20%–40%), piperacillin/tazobactam (20%–40%), sulfamethoxazole (±70%) and trimethoprim (±44%). However, for flucloxacillin with a high and variable protein binding, clinical data have shown that it is not possible to reliably estimate the unbound concentration of flucloxacillin from the total concentration. Consequently, it is advised to measure unbound flucloxacillin concentrations.^8^

This research aimed to develop and validate an UPLC–MS/MS assay for simultaneous quantification of the frequently used antibiotics ceftazidime, ciprofloxacin, flucloxacillin, unbound flucloxacillin, piperacillin, tazobactam, sulfamethoxazole and trimethoprim in human plasma. Also, *N*-acetyl sulfamethoxazole, the main metabolite of sulfamethoxazole, is quantified with this method. *N*-acetyl sulfamethoxazole has no antibacterial activity but can cause nephrotoxicity (crystalluria and haematuria) in concentrations above 75 mg/L.^9^

The assay will be applied to pharmacological research and routine TDM for any given patient that requires assessment of plasma concentrations of these antimicrobial drugs.

## Material and methods

### Chemicals and reagents

Details on the purchase of the chemicals and reagents are described in Appendix S1 (available as Supplementary data at *JAC* Online).

### Preparation of stock and working solutions, precipitation reagent, calibration standards and QCs

Detailed information can be found in Appendix S1. In short, stock and working solutions of each compound and its isotopically labelled internal standard (IS) were made independently for the preparation of eight calibration standards and six quality control (QC) samples. Calibration standards and QCs [lower limit of quantification (LLOQ), low (QCL), medium (QCM), high (QCH), extra high (QCXH) and upper limit of quantification (ULOQ)] were prepared by spiking blank EDTA plasma with working solution containing all compounds in ultrapure water.

For the measurement of protein-unbound flucloxacillin, calibration standards were made by spiking blank EDTA plasma ultrafiltrate with a stock solution containing flucloxacillin in DMSO. QCL, QCM and QCH samples were prepared in EDTA plasma, LLOQ and ULOQ samples were prepared in plasma ultrafiltrate.

Precipitation reagent for the multi-compound assay was made by spiking MeOH with an aliquot of each IS stock solution containing an isotopically labelled IS. Precipitation reagent for the assay for the determination of unbound flucloxacillin was made by spiking MeOH with an aliquot of flucloxacillin IS stock solution.

### Sample preparation

Calibration standards, QCs and patient samples were thawed at room temperature for at least 30 min, vortexed for 3 min and centrifuged for 5 min (18 620*g* or 14 000 rpm) at room temperature.

For the measurement of total concentrations an aliquot of 25 μL of the plasma sample was transferred to an Eppendorf tube. Subsequently, 75 μL precipitation reagent was added and the mixture was vortexed for 3 min. The samples were centrifuged for 5 min (18 620*g* or 14 000 rpm) at room temperature. Next, 50 μL of supernatant was transferred into a clean vial and centrifuged (1910*g* or 3000 rpm) for 5 min at 10°C.

For the measurement of protein-unbound flucloxacillin an aliquot of 500 µL of patient sample or 500 µL of QC sample was prepared in the same way as obtaining blank plasma ultrafiltrate. Next, 300 µL of precipitation reagent for unbound flucloxacillin was added to 100 µL of ultrafiltrate in a maximum recovery vial (Waters). The vials were closed with a polypropylene screw cap with silicon/PTFE septum cap and vortexed for 3 min.

### Liquid chromatography-tandem mass spectrometry

The assay was carried out using an Acquity H-class UPLC system consisting of a Quaternary Solvent Manager solvent delivery pump, a flow-through needle autosampler, a column oven and a Xevo TQ-S micro triple quadrupole mass spectrometer (Waters, Etten-Leur, the Netherlands). An aliquot of 1 μL per sample was injected onto an Acquity UPLC HSS T3 1.8 μm C18 column (100 × 2.1 mm i.d.) with an Acquity UPLC HSS T3 1.8 μm C18 guard column (5 × 2.1 mm i.d.). Column and autosampler tray temperatures were set at 40 ± 5°C and 8 ± 5°C, respectively.

After sample preparation, an aliquot of 1 μL per calibration standard, QC or patient sample was injected onto the UPLC–MS/MS. Total run time was 7 minutes per sample. Specific UPLC–MS/MS settings are described in Appendix S1 and Table S2.

### Assay validation

Validation was performed according to the EMA guideline on Bioanalytical Method Validation.^10^ In addition to the parameters mentioned in the EMA guideline, parameters relating to the ultrafiltration process for the determination of protein-unbound flucloxacillin were also validated. These included recovery of the ultrafiltration process and accuracy and precision of diluted samples after ultrafiltration.

#### Calibration curve

Calibration curves were plotted as the analyte concentration against the peak area ratio of the analyte to the IS. The curves were fitted quadratically with a *1/x* weighting for ceftazidime and *N*-acetyl sulfamethoxazole and a *1/x^2^* weighting for ciprofloxacin, flucloxacillin, unbound flucloxacillin, sulfamethoxazole, piperacillin, tazobactam and trimethoprim. According to the EMA guideline, calibration curves were accepted if for ≥75% of the calibration standards the bias fell within ±15% (within ± 20% for the LLOQ) of the nominal concentration.

#### Accuracy, precision and recovery

To determine the accuracy, within- and between-day precision for each compound, five replicates of each QC level (LLOQ, QCL, QCM, QCH, QCXH and ULOQ) were prepared and analysed during three different runs executed on three different days.

Within-day accuracy was calculated using the equation:


(1) $$\frac{\mathrm{mean}\;\mathrm{measured}\;\mathrm{concentration}\;\mathrm{within}\;a\;\mathrm{run}\;}{\mathrm{nominal}\;\mathrm{concentration}}*100\text{\%}$$

Between-day accuracy was calculated as the mean of the within-day accuracies of three different runs.

Within-day precision was calculated using the equation:


(2) $$\frac{\mathrm{SD}\;\mathrm{of}\;\mathrm{measured}\;\mathrm{concentrations}\;\mathrm{within}\;a\;\mathrm{run}\;}{\mathrm{mean}\;\mathrm{measured}\;\mathrm{concentration}\;\mathrm{within}\;a\;\mathrm{run}}*100\text{\%}$$

From a one-way analysis of variance, the mean square within groups (MS_within_), the mean square between groups (MS_between_) and the overall mean of the 15 measurements of three runs were obtained. These were used to calculate the between-day precision using the following equation:


(3) $$\begin{array}{r} \frac{\sqrt{\frac{\left(\mathrm{MSwithin}-\mathrm{Msbetween}\right)}{n}}\;}{\mathrm{overall}\;\mathrm{mean}}*100\text{\%},\;\;\\ n\;\mathrm{is}\;\mathrm{the}\;\mathrm{number}\;\mathrm{of}\;\mathrm{replicates}\;\mathrm{within}\;a\;\mathrm{run} \end{array}$$

Traditional determination of the accuracy of the ultrafiltration process of the flucloxacillin samples is not possible because the true protein-binding equilibrium remains unknown and cannot be spiked or pre-set. Alternatively, accuracy, within- and between-day precision were determined without the ultrafiltration step by quantification of the LLOQ and ULOQ in 5-fold in EDTA plasma ultrafiltrate. Second, within- and between-day precision with the ultrafiltration step were determined by analysis of the QCL, QCM and QCH EDTA plasma samples. Accuracy and precision were assessed and calculated as previously described.^11^

For accuracy, the mean concentration should be within ±15% of nominal concentration (within ±20% for the LLOQ). For precision, the CV should not exceed 15% (20% for the LLOQ).

Extraction recovery was determined in duplicate at QCL, QCM and QCH level. This was calculated by dividing the peak area of spiked blank plasma with the peak area of blank processed plasma spiked with a solution containing all analytes.

To determine whether flucloxacillin was lost during the ultrafiltration process, the recovery of the ultrafiltration process was evaluated for five replicates at each QC level as previously described.^11^ Preferably, extraction recovery was >70%.

#### Selectivity and carry-over

To determine the selectivity of the assay, processed blank EDTA plasma samples (without IS) from six individuals were individually analysed. Assessment of carry-over was performed by injecting a blank plasma sample without IS after injection of the ULOQ. This was repeated five times.

Interference and carry-over should not exceed 20% of the signal for the LLOQ and should not exceed 5% of the signal for the IS.

#### Matrix effect

The matrix effect was determined in duplicate in six individual lots of blank plasma (matrix), which were spiked with analyte and IS at concentrations of QCL and QCH. The matrix factor (MF) was calculated for each analyte and IS by calculating the ratio of the peak area in the presence of matrix to the peak area in the absence of matrix (solution of the analyte in MeOH/water). The percentage CV of the IS-normalized MF (ratio of the MF of the analyte to the MF of the IS) was calculated and should not exceed 15%.

#### Dilution integrity

Five replicate samples with a concentration of 1.5 times the ULOQ were diluted with blank EDTA plasma 2- or 4-fold, and subsequently processed and analysed.

For the assay measuring unbound flucloxacillin, five replicate samples with a concentration of 1.5 times the QCH were used.

Per dilution factor, accuracies should be within ±15% of the nominal concentration and precisions should not exceed 15%.

#### Stability

Spiked samples were tested in duplicate at four different concentrations (QCL, QCM, QCH, QCXH) for short-term stability (at room temperature and 4°C–8°C), long-term stability at −40°C and stability after three freeze (−40°C)–thaw cycles. For stability in the autosampler (8°C ± 5°C), five replicates of processed samples with concentrations at LLOQ, QCL, QCM, QCH, QCXH and ULOQ level were tested after 24 hours and 5 days.

For each compound, samples of three patients were evaluated in duplicate to assess short-term stability in EDTA whole blood and EDTA plasma, and freeze–thaw stability in EDTA plasma.

Analytes were considered stable when the mean measured concentration at each level was within ±15% (within ±20% for the LLOQ) of the nominal concentration.

Stability of the stock solutions at −40°C was evaluated when fresh stocks were prepared. Stock solutions were considered stable if the mean measured concentration at each level was within ±5% of the nominal concentration.

### Ethics

Ethical approval was obtained from the medical ethics research committee of the Radboudumc (METC East-Netherlands, Nijmegen, the Netherlands) with a waiver for informed consent for the use of anonymous drug concentration data obtained for clinical practice, for evaluation of the assay in clinical practice.

## Validation results

### Calibration curve

All calibration curves were adequately described by quadratic regression over the concentration ranges described in Table S1. All calibration standards passed the acceptance criteria.

### Accuracy, precision and recovery

Table 1 shows the within- and between-day accuracies and precisions (% CV) of the assay. All accuracies and CVs fell within the acceptance criteria. The within-day accuracy ranged from 90.0% (ceftazidime at LLOQ) to 109% (flucloxacillin at LLOQ) and between-day accuracy ranged from 93.4% (ceftazidime at LLOQ) to 108% (flucloxacillin at LLOQ). Within-day CV varied from 1.70% (sulfamethoxazole at LLOQ) to 11.2% (*N*-acetyl sulfamethoxazole at QCM) and between-day CV varied from 0.290% (tazobactam at QCL) to 5.30% (tazobactam at QCH).

**Table 1.** Accuracy and precision of the measurement of the total concentration of each analyte

	LLOQ		QCL		QCM		QCH		QCXH		ULOQ	
	Accuracy (%)	Precision (% CV)	Accuracy (%)	Precision (% CV)	Accuracy (%)	Precision (% CV)	Accuracy (%)	Precision (% CV)	Accuracy (%)	Precision (% CV)	Accuracy (%)	Precision (% CV)
Within-day (*n* = 5)												
Ceftazidime	90.0	4.51	94.2	3.55	96.6	6.31	97.0	2.58	94.1	3.55	93.8	2.36
Ciprofloxacin	95.6	3.49	97.0	2.34	103	10.4	96.8	2.32	96.9	3.92	103	3.50
Flucloxacillin	109	4.61	104	4.53	102	2.69	103	4.07	106	5.36	107	4.32
Piperacillin	105	4.41	97.8	1.76	95.4	7.63	97.0	2.39	96.4	4.64	99.3	1.82
Sulfamethoxazole	94.0	1.70	103	2.10	96.4	8.76	96.0	2.15	97.6	4.12	102	2.05
*N*-acetyl sulfamethoxazole	93.2	3.75	103	4.80	105	11.2	98.0	2.11	96.7	5.01	96.3	1.76
Tazobactam	102	2.72	97.3	6.00	98.6	10.8	106	5.30	104	6.98	104	6.07
Trimethoprim	94.8	3.44	98.5	2.76	97.9	8.88	93.7	2.15	96.5	4.21	103	3.01
Between-day (*n* = 15)												
Ceftazidime	93.4	4.02	97.3	2.54	97.7	^a^	97.5	^a^	95.2	0.300	95.5	1.67
Ciprofloxacin	98.8	3.21	99.8	2.61	100	^a^	99.3	2.04	98.2	0.780	100	2.46
Flucloxacillin	108	^a^	102	0.610	101	1.26	101	1.07	104	0.940	106	^a^
Piperacillin	101	2.79	99.2	1.06	96.2	^a^	98.9	1.45	97.2	^a^	99.6	^a^
Sulfamethoxazole	98.3	3.97	101	2.56	98.1	^a^	97.8	1.84	98.7	0.690	101	0.490
*N*-acetyl sulfamethoxazole	96.1	3.50	101	1.98	102	^a^	100	1.79	98.1	^a^	97.2	0.700
Tazobactam	101	1.33	99.7	0.290	99.2	^a^	101	5.30	99.8	2.62	101	^a^
Trimethoprim	97.5	2.72	100	0.990	99.1	^a^	95.4	1.50	96.9	^a^	102	1.20

^a^No value could be calculated for the between-day precision if MS_between_ was higher than MS_within_ causing (MS_within_ − MS_between_) to have a negative value, see Equation (3). Since the square root of a negative value is mathematically not possible, calculation of the between-day precision was not possible.

Table 2**(a)** shows the results of the accuracy and precision of the LLOQ and ULOQ samples without the ultrafiltration process. Table 2**(b)** presents the results of the precision of the ultrafiltration process with QCL, QCM and QCH samples. The maximum within-day variation coefficient was 3.23% (QCH) and the maximum between-day precision variation coefficient was 2.93% (QCM). All parameters fell within the predefined acceptance criteria.

**Table 2.** (a) Accuracy (without ultrafiltration) and (b) precision (with and without ultrafiltration) of the unbound flucloxacillin concentration

	Concentration spiked in ultrafiltrate (mg/L)	Within-day accuracy (%)	Between-day accuracy (%)	Within-day precision (% CV)	Between-day precision (% CV)
(a)					
LLOQ	0.0980	106	105	7.11	^a^
ULOQ	49.0	103	102	1.92	0.800

	Concentration spiked Plasma (mg/L)	Measured unboundconcentration (mg/L)	Accuracy (%)	Within-day precision (% CV)	Between-day precision (% CV)
(b)					
QCL	1.00	0.281	^b^	2.85	^a^
QCM	10.0	6.10	^b^	2.50	2.93
QCH	100	26.2	^b^	3.23	2.88

^a^No value could be calculated for the between-day precision if MS_between_ was higher than MS_within_ causing (MS_within_ − MS_between_) to have a negative value. Since the square root of a negative value is mathematically not possible, calculation of the between-day precision was not possible.

^b^Determining the accuracy of the ultrafiltration process is not possible, since the nominal concentration of unbound flucloxacillin cannot be set.

The lowest mean extraction recovery was 77.0% and the highest mean extraction recovery was 105%. Extraction recovery results are shown in Table S3. The extraction recovery was consistent (SD ≤ 15%) and high (>70%) over the validated range.

Recovery of the ultrafiltration process was deemed acceptable; within 85%–115% for all measured samples and percentage CVs for each concentration level were ≤15%. Results are shown in Table 3.

**Table 3.** Recovery of the ultrafiltration process

	QCL (%)	QCM (%)	QCH (%)
Replicate 1	84.4	102	101
Replicate 2	89.2	88.4	107
Replicate 3	82.7	98.1	90.9
Replicate 4	103	106	96.4
Replicate 5	89.2	103	101
Mean	89.7	99.5	99.1
SD	8.08	6.82	5.82
% CV	9.00	6.86	5.87

### Selectivity and carry-over

Chromatograms of blank plasma and spiked plasma at the LLOQ are shown in Figure S1. Results regarding selectivity were all within acceptance criteria (Table S4). Carry-over results were all within acceptance criteria (Table S5).

### Matrix effect

All analytes had a CV of the IS-normalized MF <15% (Table S6).

### Dilution integrity

Per dilution factor, accuracies were all within ±15% of the nominal concentration and precisions did not exceed 15% of the nominal concentration (Table S7).

### Stability

Results of the stability tests at various conditions are shown in Table 4. In addition to the data in Table 4, spiked samples and patient samples were shown to be stable after three freeze–thaw cycles at −40°C for all analytes. Processed samples were stable in vials in the autosampler (4°C–8°C) for at least 5 days.

**Table 4.** Stability data at various conditions in spiked EDTA plasma, patient EDTA plasma and patient EDTA blood

	Spiked EDTA plasma			Patient EDTA plasma		Patient EDTA blood	
	Room temperature	4°C–8°C	−40°C	Room temperature	4°C–8°C	Room temperature	4°C–8°C
Ceftazidime	At most 5 hours	At most 2 days	At least 2 years	At most 6 hours	At most 3 days	At most 24 hours	At least 6 days
Ciprofloxacin	At least 6 days	At least 6 days	At least 2 years	At least 5 days	At least 5 days	At least 3 days	At most 20 hours
Flucloxacillin	At most 5 hours	At most 2 days	At least 2 years	At most 24 hours	At most 3 days	At least 3 days	At least 3 days
Piperacillin	At most 5 hours	At most 1 day	At least 2 years	At most 5 hours	At most 2 days	At most 24 hours	At least 5 days
Sulfamethoxazole	At least 6 days	At least 6 days	At least 2 years	At least 5 days	At least 5 days	At most 5 hours	At most 3 days
*N*-acetyl sulfamethoxazole	At least 6 days	At least 6 days	At least 2 years	At least 5 days	At least 5 days	At most 3 hours	At most 24 hours
Tazobactam	At most 5 hours	At most 1 day	At least 2 years	At most 24 hours	At least 5 days	At most 24 hours	At least 5 days
Trimethoprim	At least 6 days	At least 6 days	At least 2 years	At least 5 days	At least 5 days	At least 5 days	At least 5 days

Stock solutions of ceftazidime, ciprofloxacin, piperacillin, sulfamethoxazole, *N*-acetyl sulfamethoxazole and trimethoprim were stable for at least 9 months at −40°C. Flucloxacillin stock solution was stable for at least 34 months at −40°C and tazobactam stock solution was stable for at least 38 months at −40°C. Working solutions were stable for at least 31 months at −40°C.

## Application to research and clinical practice

The assay has been used to analyse patient samples for research purposes, such as the investigation of the pharmacokinetics of piperacillin and tazobactam and unbound and total flucloxacillin in critically ill patients.^12,13^

**Figure 1.**
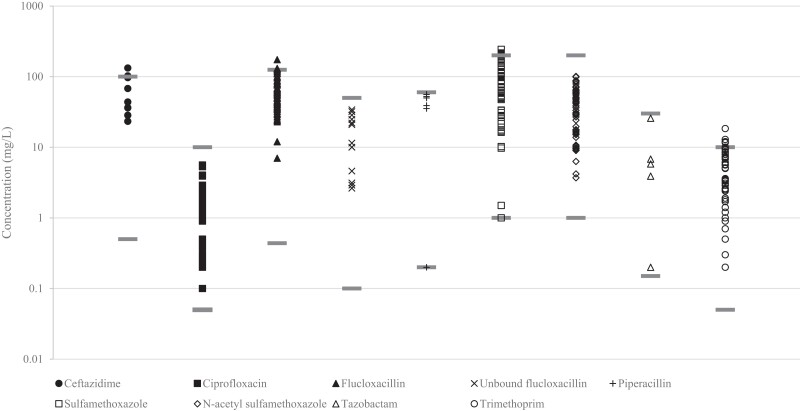
Measured antibiotic concentrations in the TDM programme in our hospital (*n* = 356). The dark grey horizontal lines indicate the validated range of the corresponding compound.

Both validated assays are now used for routine TDM in our hospital. Results from analysis of patient samples from February 2019 to March 2022 are shown in Figure 1. Of all patient samples analysed for routine clinical care (*n* = 356) 88.2% fell within the validated range, 2.81% was below the LLOQ and 8.99% was above the ULOQ.

## International quality control programme participation

Reliability of the results of the presented assays is evaluated by participation in an external QC programme by the Dutch Foundation of Quality Assessment of Medical Laboratories (Stichting Kwaliteitsbewaking Medische Laboratoriumdiagnostiek, SKML).^14^ All compounds analysed by the described method are included in this programme, which is run twice a year. From 2019 until the first quarter of 2023, our laboratory has participated 10 times. In this programme, the acceptance criterion for accuracy is that the measured concentration should be within ±20% of the true concentration. For unbound flucloxacillin there is no true concentration, therefore a consensus value is used.

Measured concentrations were all within ±20% of the true value, except for one out of 18 measurements of ciprofloxacin and two out of 18 measurements of ceftazidime (Table 5).

**Table 5.** Results of the participation of the QC programme of antimicrobial drugs by the SKML

	Number of measurements	Mean measured concentration relative to true value (%) [range]
Ceftazidime	18	109 [99.4–122]
Ciprofloxacin	18	108 [97.6–121]
Flucloxacillin	18	106 [96.0–120]
Unbound flucloxacillin	10^a^	137 [96.4–163]^b^
Piperacillin	18	108 [98.5–120]
Sulfamethoxazole	18	100 [96.7–112]
*N*-acetyl sulfamethoxazole	18	103 [94.7–116]
Tazobactam	2^c^	103 [98.7–107]
Trimethoprim	18	98.5 [91.4–115]

^a^Unbound flucloxacillin has been part of the programme since 2021.

^b^Mean measured concentration relative to the consensus value. For our assay, ultrafiltration was performed at a temperature of 37°C.

^c^Tazobactam was only included in the programme in 2019.

It was observed that with our assay, higher unbound flucloxacillin concentrations were reported than some other participating laboratories. In-depth research on this matter shows that this was caused by other laboratories not taking physiological conditions (i.e. ultrafiltration at 37°C) for the protein unbound–bound equilibrium into account.^15^ When comparing our results to results of others performing ultrafiltration at 37°C, they were in line with the consensus values.

## Discussion and conclusion

We successfully developed and validated UPLC–MS/MS assays for quantification of total concentrations of ceftazidime, ciprofloxacin, flucloxacillin, piperacillin, sulfamethoxazole, *N*-acetyl sulfamethoxazole, tazobactam, trimethoprim and unbound concentrations of flucloxacillin in human plasma. Results of an external QC programme indicate that the assays provide reliable results. The assays have been used for research and TDM purposes.

Other analytical methods measuring plasma concentrations of multiple antibiotics have been described, nevertheless none of these measures the same combination of antibiotics as with this assay.^16–22^ This combination of antibiotics was chosen based on frequency of prescription in critically ill patients, where optimized dosing is most important. Second, for these antibiotics, large interpatient variability in pharmacokinetics and target exposures are described. Third, for these antibiotics, no alternative assay was available in the region our hospital is located. Next to the unique combination of frequently prescribed antibiotics our assay has multiple other advantages. A low volume of plasma is required (25 μL), and the run time (7 minutes) is relatively short compared to other published methods, enabling its use in daily clinical practice.^16–22^ The use of stable isotopically labelled internal standards for all compounds, either deuterated or labelled with ^13^C or ^15^N, makes it possible to compensate for matrix effects or incomplete recoveries.^23^ Furthermore, the chosen calibration range makes the assay very suitable for TDM, since low and high concentrations can be present in (critically ill) patients.

To ensure reliable results of the described assay we participate in an international QC programme. The results showed that our assay is robust and reliable, which is of paramount importance to enable the use of this assay in clinical practice. Moreover, participation resulted in the investigation of observed differences in reported unbound flucloxacillin concentrations, that were most likely to be based on differences in ultrafiltration temperature. This finding is of importance to ensure adequate unbound flucloxacillin measurement not only in our laboratory, but in all laboratories measuring unbound flucloxacillin concentration to improve patient care.

Stability tests showed the instability of β-lactam antibiotics at room temperature. To prevent decomposition, solutions and samples should be either stored in the refrigerator or freezer (see Table 4 for validated storage periods). Furthermore, a cooled autosampler should be used, and samples should be extracted shortly before analysis.

Analysis of patient samples showed that 88.2% fell within the range of the assay. Since dilution integrity was demonstrated during the validation of this assay, all samples with a concentration above the calibration range (8.99%) could be reliably measured when diluted 2–4 times. The LLOQ for each antibiotic was set as low as clinically relevant; for the patient samples that measured below the LLOQ (2.81%), quantification of the exact concentrations would not affect clinical decision making.

## Supplementary Material

dkae047_Supplementary_Data

## Supplementary data

Appendix S1, Figure S1 and Tables S1–S7 are available as Supplementary data at *JAC* Online.
