# Supplementary material for: Development and validation of an UPLC–MS/MS assay for the simultaneous quantification of seven commonly used antibiotics in human plasma and its application in therapeutic drug monitoring
Source: J Antimicrob Chemother. 2024 Feb 28;79(4):883–90. doi: 10.1093/jac/dkae047 (PMC10984926; doi:10.1093/jac/dkae047)
Supplement: dkae047_Supplementary_Data [file dkae047_supplementary_data.docx]

**Supplementary material**

Appendix 1

*Chemicals and reagents*

Ceftazidime pentahydrate, ceftazidime-*d*5, ciprofloxacin-*d*8, flucloxacillin Na, flucloxacillin Na ^13^C_4_, piperacillin, piperacillin-*d*5, sulfamethoxazole, sulfamethoxazole-*d*4, *N*-acetyl sulfamethoxazole, *N*-acetyl sulfamethoxazole-*d*4, tazobactam Na ^15^N_3_, and trimethoprim-*d*3 were purchased from Toronto Research Chemicals (Toronto, Canada). Tazobactam and trimethoprim were purchased from Sigma-Aldrich (Amsterdam, The Netherlands), ciprofloxacin HCl was purchased from Carbosynth Limited (Berkshire, United Kingdom).

Ultrapure water was obtained using a Purelab flex 4 system from Veolia (Ede, the Netherlands). Acetonitrile hypergrade, formic acid for U-LC/MS, methanol for U-LC/MS and DMSO were purchased from Merck (Darmstadt, Germany). 0.1M HCl solution was prepared with 37% HCl from Merck (Darmstadt, Germany).

Drug-free EDTA whole blood was obtained from Sanquin (Nijmegen, the Netherlands) and centrifuged for 5 min at 1900*g* at room temperature to obtain EDTA plasma. EDTA plasma was pooled, except for the determination of the selectivity and matrix effect, and stored at -20°C until use.

To obtain blank plasma ultrafiltrate, 500 µL blank human EDTA plasma was pipetted onto the ultrafiltration filters (Centrifree YM-30 ultrafiltraton filter device, Milipore B.V., Amsterdam, the Netherlands). It was equilibrated for 60 minutes at 1*g*/41°C to reach a sample temperature of 37°C and then ultrafiltrated by centrifuging for 20 minutes at 1650*g* at 41°C.

*Preparation of stock and working solutions and precipitation reagent*

Stock solutions for quality control samples (QCs) and calibration standards were prepared independently at a concentration of ca. 5 mg/mL (ceftazidime, flucloxacillin, piperacillin, tazobactam, trimethoprim) and ca. 25 mg/mL (sulfamethoxazole and *N*-acetyl sulfamethoxazole) in DMSO. Stock solutions of ciprofloxacin were prepared at a concentration of ca. 5 mg/mL in 0.1M HCl.

Working solutions for calibration standards and QCs were prepared by diluting stock solutions with ultrapure water to concentrations of 2000 mg/L for sulfamethoxazole and *N*-acetyl sulfamethoxazole, 1000 mg/L for ceftazidime, 600 mg/L for piperacillin, 300 mg/L for tazobactam, 1250 mg/L for flucloxacillin, and 100 mg/L for trimethoprim and ciprofloxacin. Internal standard (IS) stock solutions were prepared at a concentration of ca. 1 mg/mL in DMSO, except for ciprofloxacin which was prepared in 0.1M HCl.

Working solutions for calibration standards and QCs for the measurement of protein-unbound flucloxacillin were prepared in concentrations of 0, 0.300, 1.00, 3.00, 10.0, 30.0, 100 mg/L by diluting the flucloxacillin stock solution with DMSO.

Precipitation reagent was obtained by mixing 30 ml MeOH with an aliquot of IS stock solution of each compound; 15 μl ceftazidime-IS, 30 μl ciprofloxacin-IS, 90 μl flucloxacillin-IS, 15 μl piperacillin-IS, 2 μl sulfamethoxazole-IS, 2 μl *N*-acetyl sulfamethoxazole-IS, 150 μl tazobactam-IS, 4 μl trimethoprim-IS.

Precipitation reagent for the measurement of protein-unbound flucloxacillin was made by mixing 45 µL flucloxacillin-IS with 45 mL MeOH.

All solutions were stored at -40°C until use.

*Preparation of calibration standards and QCs*

Eight calibration standards were prepared by spiking blank EDTA plasma with working solution for the calibration curve. QC samples (lower limit of quantification (LLOQ), low, medium, high, extra high (QCXH), and upper limit of quantification (ULOQ)) were prepared by spiking blank EDTA plasma with working solution for QCs. Concentrations of the calibration standards and QCs are shown in table S1.

Calibration standards for the measurement of protein-unbound flucloxacillin were prepared freshly on the day of analysis by diluting working solution for measurement of protein-unbound flucloxacillin 10-fold with blank EDTA plasma ultrafiltrate. QCL, QCM, and QCH samples were prepared by spiking blank EDTA plasma with flucloxacillin stock solutions for QCs, resulting in a concentration of 1.00 mg/L, 10.0 mg/L, and 100 mg/L, respectively. QC samples underwent the ultrafiltration process as described previously. LLOQ and ULOQ samples were prepared in plasma ultrafiltrate at concentrations of 0.100 mg/L and 50.0 mg/L, respectively.

All calibration standards and QCs were stored at -40°C until use.

*Liquid chromatography-tandem mass spectrometry settings*

The initial mobile phase composition was 95% mobile phase A (0.1% formic acid in water (v/v)) and 5% mobile phase B (0.1% formic acid in acetonitrile (ACN) (v/v)), which changed linearly to 30% A and 70% B at 4.00 min. From 4.01 to 7.00 min the mobile phase composition returned linearly to starting conditions. For the measurement of protein-unbound flucloxacillin, mobile phase B was substituted for mobile phase C (1% formic acid in acetonitrile (*v/v*)).

The flow rate was 0.500 mL/min with a total runtime of 7.00 min. The needle was washed for 3 seconds pre- and post-injection with a solution of water/ACN/formic acid 40/60/1 (v/v/v).

Analytes were measured in positive electrospray ionization mode using multiple reaction monitoring (MRM). The capillary voltage was 1.40 kV, source temperature was 150°C, desolvation temperature was 500°C, desolvation gas flow was 950 L/h (nitrogen) and cone gas flow was 20 L/h (nitrogen). The nebulizer gas used was nitrogen, the collision gas used was argon. MRM transition, cone voltage and collision energy were optimized using IntelliStart (Waters). Optimized MRM transition, cone voltage and collision energy are shown in table S2. Data was processed using Masslynx software version 4.1 (Waters). Quantification was performed using TargetLynx software (Waters).

**Supplementary – Figure**

1.82 min

547.07 > 467.97

IS 1.81 min

IS 552.07 > 468.07

Response

Response

**Figure S1**. Multiple reaction monitoring (MRM) chromatograms of an LLOQ sample, IS sample and blank sample (ST 0). Also shown are the retention time (RT) and MRM transition of the LLOQ, and the RT and MRM transition of the IS, respectively.

**Supplementary – Tables**

**Table S1.** Concentrations of calibration standards and quality control samples (mg/L)

|  | **Ceftazidime** | **Ciprofloxacin** | **Flucloxacillin** | **Piperacillin** | **Unbound flucloxacillin** | **Sulfamethoxazole** | ***N*-acetyl sulfamethoxazole** | **Tazobactam** | **Trimethoprim** |
| --- | --- | --- | --- | --- | --- | --- | --- | --- | --- |
| **Cal 1** | 0.500 | 0.0500 | 0.438 | 0.200 | 0.0300 | 1.00 | 1.00 | 0.150 | 0.0500 |
| **Cal 2** | 1.00 | 0.100 | 0.625 | 0.300 | 0.100 | 2.00 | 2.00 | 0.300 | 0.100 |
| **Cal 3** | 2.00 | 0.200 | 1.25 | 0.600 | 0.300 | 4.00 | 4.00 | 0.600 | 0.200 |
| **Cal 4** | 5.00 | 0.500 | 2.50 | 1.20 | 1.00 | 10.0 | 10.0 | 1.50 | 0.500 |
| **Cal 5** | 10.0 | 1.00 | 6.25 | 3.00 | 3.00 | 20.0 | 20.0 | 3.00 | 1.00 |
| **Cal 6** | 50.0 | 5.00 | 12.5 | 6.00 | 10.0 | 100 | 100 | 15.0 | 5.00 |
| **Cal 7** | 100 | 10.0 | 62.5 | 30.0 | - | 200 | 200 | 30.0 | 10.0 |
| **Cal 8** | * | * | 125 | 60.0 | - | * | * | * | * |
| **LLOQ** | 0.500 | 0.0500 | 0.438 | 0.200 | 0.100 | 1.00 | 1.00 | 0.150 | 0.0500 |
| **QCL** | 0.800 | 0.0800 | 1.00 | 0.480 | 1 | 1.60 | 1.60 | 0.240 | 0.0800 |
| **QCM** | 4.00 | 0.400 | 5.00 | 2.40 | 10.0 | 8.00 | 8.00 | 1.20 | 0.400 |
| **QCH** | 40.0 | 4.00 | 50.0 | 24.0 | 100 | 80.0 | 80.0 | 12.0 | 4.00 |
| **QCXH** | 80.0 | 8.00 | 100 | 48.0 | - | 160 | 160 | 24.0 | 8.00 |
| **ULOQ** | 100 | 10.0 | 125 | 60.0 | 50.0 | 200 | 200 | 30.0 | 10.0 |

* Calibration standard 8 only included flucloxacillin and piperacillin

- Unbound flucloxacillin only had six calibrators, and no QCXH. The QCL, QCM and QCH concentrations shown, are before ultrafiltration.

**Table S2.** Mass spectrometry parameters

|  | MRM transition (Da) | Cone voltage (V) | Collision energy (eV) | Retention time (min) |
| --- | --- | --- | --- | --- |
| Ceftazidime | 547.07 > 467.97 | 50 | 12 | 1.82 |
| Ceftazidime-*d*5 | 552.07 > 468.07 | 50 | 10 | 1.81 |
| Ciprofloxacin | 332.10 > 245.07 | 64 | 22 | 2.23 |
| Ciprofloxacin-*d*8 | 339.98 > 248.99 | 64 | 24 | 2.22 |
| Flucloxacillin | 453.83 > 160.06 | 58 | 16 | 4.25 |
| Flucloxacillin ^13^C_4_ | 457.87 > 197.94 | 58 | 36 | 4.25 |
| Piperacillin | 518.01 > 143.00 | 56 | 16 | 3.35 |
| Piperacillin-*d*5 | 523.11 > 148.03 | 56 | 16 | 3.33 |
| Sulfamethoxazole | 253.96 > 155.97 | 38 | 14 | 2.94 |
| Sulfamethoxazole-*d*4 | 257.90 > 95.99 | 38 | 24 | 2.93 |
| *N*-acetyl sulfamethoxazole | 298.80 > 134.80 | 64 | 28 | 3.04 |
| *N*-acetyl sulfamethoxazole-*d*4 | 300.96 > 138.84 | 64 | 26 | 3.03 |
| Tazobactam | 300.82 > 98.79 | 48 | 24 | 1.63 |
| Tazobactam Na ^15^N_3_ | 303.82 > 101.80 | 48 | 14 | 1.63 |
| Trimethoprim | 291.11 > 230.07 | 60 | 22 | 2.15 |
| Trimethoprim-*d*3 | 293.93 > 122.91 | 60 | 24 | 2.13 |

MRM: Multiple Reaction Monitoring

**Table S3.** Extraction recovery results

|  | **QCL** | **QCM** | **QCH** | **Mean** | **SD** |
| --- | --- | --- | --- | --- | --- |
| **Ceftazidime** | * | * | * | * | * |
| **Ciprofloxacin** | 105 | 106 | 103 | 105 | 1.00 |
| **Flucloxacillin** | 81.0 | 72.0 | 89.0 | 81.0 | 8.00 |
| **Piperacillin** | 113 | 99.0 | 92.0 | 102 | 10.0 |
| **Sulfamethoxazole** | 88.0 | 87.0 | 95.0 | 90.0 | 4.00 |
| ***N*-acetyl sulfamethoxazole** | 116 | 95.0 | 95.0 | 102 | 12.0 |
| **Tazobactam** | 81.0 | 71.0 | 78.0 | 77.0 | 5.00 |
| **Trimethoprim** | 93.0 | 88.0 | 94.0 | 91.0 | 3.00 |

All results are shown as percentages.

* Extraction recovery of ceftazidime was nonquantifiable, due to very poor solubility in MeOH/water and MeOH/NaCl 0.9%. SD = standard deviation.

**Table S4.** Selectivity results

|  | **Mean* response as % of the LLOQ response**  **[range]** | **Mean* response as % of the IS response**  **[range]** |
| --- | --- | --- |
| **Ceftazidime** | 0.168 [0.0870 – 0.280] | 0.0241 [0.00578 – 0.0559] |
| **Ciprofloxacin** | 0.508 [0.0397 – 0.825] | 0.151 [0.126 – 0.206] |
| **Flucloxacillin** | 0.539 [0.147 – 1.18] | 0.0659 [0.0304 – 0.122] |
| **Piperacillin** | 0.434 [0.317 – 0.582] | 0.0492 [0.0385 – 0.0767] |
| **Sulfamethoxazole** | 0.303 [0.226 – 0.453] | 0.354 [0.192 – 0.600] |
| ***N*-acetyl sulfamethoxazole** | 0.133 [0.0736 – 0.177] | 0.582 [0.474 – 0.677] |
| **Tazobactam** | 0.352 [0.188 – 0.634] | 0.905 [0.688 – 1.41] |
| **Trimethoprim** | 0.181 [0.110 – 0.248] | 0.0678 [0.0382 – 0.0965] |

* n = 6 samples

**Table S5.** Carry-over results

|  | **Mean* response as % of LLOQ response**  **[range]** | **Mean* response as % of IS response**  **[range]** |
| --- | --- | --- |
| **Ceftazidime** | 1.20 [0.996 – 1.37] | 0.0391 [0.0299 – 0.0463] |
| **Ciprofloxacin** | 12.4 [11.1 – 13.8] | 0.195 [0.169 – 0.234] |
| **Flucloxacillin** | 5.59 [3.30 – 9.31] | 0.0426 [0.0213 – 0.0638] |
| **Piperacillin** | 7.77 [6.94 – 8.18] | 0.0584 [0.0480 – 0.0704] |
| **Sulfamethoxazole** | 3.44 [3.24 – 3.65] | 0.376 [0.264 – 0.591] |
| ***N*-acetyl sulfamethoxazole** | 2.94 [1.09 – 3.95] | 0.509 [0.406 – 0.590] |
| **Tazobactam** | 1.57 [1.32 – 1.88] | 0.969 [0.103 – 1.34] |
| **Trimethoprim** | 2.55 [2.32 – 2.83] | 0.0959 [0.0731 – 0.117] |

* n = 5 samples

**Table S6.** Matrix effect results

|  | **QCL**  **(% CV of IS-normalised MF)** | **QCH**  **(% CV of IS-normalised MF)** |
| --- | --- | --- |
| **Ceftazidime** | 3.96 | 11.5 |
| **Ciprofloxacin** | 3.88 | 14.3 |
| **Flucloxacillin** | 3.63 | 7.48 |
| **Piperacillin** | 3.92 | 10.7 |
| **Sulfamethoxazole** | 3.39 | 10.4 |
| ***N*-acetyl sulfamethoxazole** | 3.48 | 11.1 |
| **Tazobactam** | 5.02 | 10.4 |
| **Trimethoprim** | 3.39 | 9.91 |

CV = variation coefficient

**Table S7.** Dilution integrity

|  | **Dilution factor 2** | |  | **Dilution factor 4** | |
| --- | --- | --- | --- | --- | --- |
|  | **Accuracy (%)** | **Within-day precision (%)** |  | **Accuracy (%)** | **Within-day precision (%)** |
| **Ceftazidime** | 97.5 | 1.64 |  | 97.5 | 1.75 |
| **Ciprofloxacin** | 99.8 | 1.52 |  | 96.6 | 2.24 |
| **Flucloxacillin** | 97.3 | 1.19 |  | 97.4 | 2.45 |
| **Unbound flucloxacillin** | 101 | 1.45 |  | 102 | 0.839 |
| **Piperacillin** | 98.3 | 1.74 |  | 97.6 | 1.19 |
| **Sulfamethoxazole** | 97.0 | 1.77 |  | 95.4 | 1.40 |
| ***N*-acetyl sulfamethoxazole** | 97.0 | 1.59 |  | 96.6 | 2.52 |
| **Tazobactam** | 98.1 | 2.91 |  | 98.8 | 2.92 |
| **Trimethoprim** | 102 | 2.42 |  | 97.3 | 1.13 |
